# AI for identifying social norm violation

**DOI:** 10.1038/s41598-023-35350-x

**Published:** 2023-05-19

**Authors:** Yair Neuman, Yochai Cohen

**Affiliations:** 1 The Functor Lab, Department of Cognitive and Brain Science, Ben-Gurion University of the Negev, 84105 Beersheba, Israel; 2 Ben-Gurion University of the Negev, Beer-Sheba, Israel

**Keywords:** Computational science, Social evolution

## Abstract

Identifying social norms and their violation is a challenge facing several projects in computational science. This paper presents a novel approach to identifying social norm violations. We used GPT-3, zero-shot classification, and automatic rule discovery to develop simple predictive models grounded in psychological knowledge. Tested on two massive datasets, the models present significant predictive performance and show that even complex social situations can be functionally analyzed through modern computational tools.

## Introduction

The APA Dictionary of Psychology defines social norms^1^ as socially determined standards that indicate typical and proper behaviors in a given social context. Some norms are universal (e.g., it is wrong to murder), some are contextual within the culture (e.g., it is not legitimate to expose your body in public, but it is legitimate to expose it on a nudist beach), and some are highly particular (e.g., in the United States it is expected to tip bartenders). Moreover, the dictionary suggests that, whether implicitly or implicitly, the norms proscribe actions that should be avoided as they violate a social norm (i.e., social norm violation).

Although some basic social norms seem universal, they are expressed with cultural variations^2^, as sometimes observed in cross-cultural encounters, such as those that appear in the black comedy mockumentary Borat^3^.

While social norms and their violations have been intensively studied in psychology and the social sciences^4,5^ the *automatic identification* of social norms and their violation is an open challenge that may be highly important for several projects, such as the engineering of digital interpreters supporting cross-cultural interactions and DAPRA’s computational cultural understanding program^6^ that funded the current project. It is an open challenge because we first have to identify the features/signals/variables indicating that a social norm has been violated. So far, the operational definition of theoretically grounded features is a task that has not been accomplished. For example, arriving at your office drunk and dirty is a violation of a social norm among the majority of working people. However, “teaching” the machine/computer that such behavior is a norm violation is far from trivial. For classifying behavior as violating a social norm, the machine should learn that certain measurable variables/features signal that a norm has been violated. In this sense, our paper aims to bridge the gap between social sciences and data science by engineering simple and theoretically grounded models that can successfully classify cases that involve norm violation.

This paper presents a novel approach to identifying social norm violations. The models developed in this paper rely on state-of-the-art language models such as GPT3^7,8^ NLI-based Zero Shot Text Classification^9,10^ and automatic rule discovery^11^, but are also grounded in deep theoretical understanding of human psychology and social emotions. It must be explained in a nutshell that we

1. use GPT-3 to support the identification of top-level categories of social norm violation in a bottom-up manner,
2. use NLI-based zero shot text classification, as it is an approach relevant for situations where big datasets are not available for training a model, and
3. use automatic rule discovery because we seek to identify the simplest possible models that can be understood by social scientists while avoiding the black-box complexification of some models.

We test our models on two massive datasets of short texts. The results provide strong empirical support for the validity of the models and also support the potential benefits of features’ design through deep domain expertise. Moreover, the results support the important role of social emotions in signaling norm violation and point to their future analysis and use in understanding and detecting norm violation.

## Norms, norm violation, and social emotions

Social norms cover many behavioral and moral norms that may vary across different groups and levels of granularity, from analyzing human society to personal norms. There seems to be an underlying universality about social norms^12^ where it was found, according to the “morality-as-cooperation” theory that the moral valence of seven cooperative behaviors is uniformly positive in 60 different cultures. Regardless of this universality, one may wonder whether the proposed methodology based on culturally specific textual corpora can generate universal conclusions. Our response to such a possible critique is twofold. First, we have a limited purpose which is mainly methodological: To develop and test an AI-based methodology that can identify the *violation* of social norms rather than the norms themselves. This methodology is generic and can be potentially adapted to different cultures. We do not intend to produce universal findings nor develop a methodology for identifying social norms in different cultures. We focus on identifying violations as we found that while norms may significantly vary across cultures, the repertoire of emotions used to respond to these violations is limited enough. As social emotions and their dynamic social aspect may be successfully studied across cultures, such as American-English and Chinese-Mandarin^13^, a constructive strategy for identifying the violation of social norms is to focus on a limited set of social emotions signaling the violation.

Let us elaborate on this important idea. As the number of social norms may be enormous, a simple and natural way of learning norms is through a limited number of social emotions that are evolutionary grounded and deeply associated with a universal valuation system of human beings^14^.

For example, when people feel shame, embarrassment, or regret, it is hypothesized that they acknowledge the violation of a social norm. Therefore, the violation of a norm is accompanied and signaled by social emotions that have an important function in the “recalibration of social evaluation in the minds of self and others”^15^^, P. 292^. These emotions are universal, although their particular expression may be culturally grounded. As explained by Billig about embarrassment^16^^, P. 219^: “Embarrassment, then, can be seen to possess a universal role in supporting the moral order of everyday life, whatever the nature of moral order”. The association between embarrassment and social norm violation is empirically supported^17^, as well as the association between norm violation and other negative social emotions, such as shame, guilt, and regret, that were shown to be consequential to norm violation^18^. These consequential social emotions seem to be evolutionary grounded in mechanisms of social devaluation^19^ and have a clear function in supporting cooperation^20^. It is therefore hypothesized that certain social emotions may indicate a social norm violation and may be used for this paper’s main task, which is *the classification of cases involving norm violation/confirmation*.

Inspired by the abovementioned theorization, the outlines of the paper are:

- First, using zero-shot classification, we tested our ability to automatically identify social emotions in short textual data.
- Next, we used GPT-3 for generating synthetic data and identifying violated social norms through human domain expertise.
- Relying on the outcome of GPT-3, we identified a high-level taxonomy of norms represented by ten top-level categories.
- In the third phase, we used a massive dataset of textual data, and measured social emotions and norm violations. We used features measuring social emotions, norm violation, and two other simple features for automatic rule discovery.
- Using this approach, we identified seven simple models (i.e., mathematical functions/rules) that can be used for classifying cases involving norm violation/confirmation.
- In the two major experiments, we used simple models and applied them to classify norm violations in two other massive datasets. Finally, we test our ability to identify which norm has been violated.

## Using zero-shot classification for identifying social emotions

This paper relies on zero-shot classification for identifying norm violations. Zero-shot classification addresses classification as a form of natural language inference (NLI). The task is to compute the probability that each class label can be automatically inferred/entailed from a textual premise. For instance, given the text “John failed in his studies” and a two-label class {bad, good}, the zero-shot classifier that we used in this study^9^^, accessed August, 2022^ inferred that the label “bad” is more reasonably entailed from the text (with a confidence level of 99%) than the label “good” (1% confidence). It is important to understand that a zero-shot classification predicts classes that the model didn’t “see” during its learning phase, which is why it is titled “zero-shot”. Zero-shot classification involves a deep neural network that has already been trained. We as users, don’t train the classifier but use it for classification. We insert a text as an input, several words (or higher textual units) as “labels” and ask the machine to classify the input to each label. Here is another example. We use as input the following sentence: “I had a car accident”. As labels, we use two words, “good” and “bad”. The classifier “guesses” that the correct label is “bad” with a probability of 0.823. While zero-shot classification usually involves units of complete semantics (e.g. sentence), we have decided, similarly to^21^, to use as class labels the class names only. We wanted to avoid the complexity of forming whole utterances and focus on the simplest possible labels. We are currently experimenting with the engineering of class labels composed of semantic composites such as sentences and have some powerful results supporting the methodology^22^.

In this paper, we have mainly used two sets of features/variables: (1) social emotions and (2) social norms. We first tested the performance of a zero-shot classifier in identifying five basic social emotions.

## Method

### Using zero-shot classification for identifying social emotions

To test the zero-shot classifier's performance, we adopted 25 short scenarios used by^23^ to measure social emotions. For each scenario, we produced the classification scores of the five social emotions used in their study: pride, gratitude, guilt, anger, and sadness. In other words, for each scenario, we produced five scores using the zero-shot classifier. We identified the emotion scored at the top by the classifier and compared it to the emotion scored at the top by the human subjects in^23^. We hypothesized that if the zero-shot classification may label the emotion in the text in agreement with the dominant emotion identified by the human judges in^23^, then the classifier can be used for successfully identifying social emotions in short texts.

### Results of testing the zero-shot classification in identifying social emotions

We identified the social emotion that on average scored the highest by the human judges in^23^. Next, we applied the zero-shot classifier that produced five scores for the five social emotions and identified the emotion scored highest by the classifier. The baseline for predicting social emotion in the scenario under full ignorance is *p* = 0.2, as for each scenario, the classifier should arbitrarily guess one of five social emotions. However, the zero-shot classifier’s top-identified emotion was identical to the human subjects’ top-identified emotion in 64% of the cases. Given the assumption of full ignorance and the classifier’s random guess, the Binomial distribution shows that such a level of success in prediction is highly unlikely (*p* = 0.0001797). This result provides preliminary support for the classifier’s ability to identify social emotions in short textual data, even if we use single emotion words as class labels. We want to clarify that the 64% score is not the score of correct answers but the percentage of cases in which the zero-shot classifier identified the same label as the *leading* label produced by the human raters in^23^. The human raters in^23^ are, therefore, our gold standard of truth for testing our system. In this context, we should ask how likely it is to produce a random guess of 64%. For testing this hypothesis, we used the Binomial distribution and showed that gaining such a level of success by chance is highly unlikely, with a probability of 0.0002 (rounded).

### Identifying categories of social norms

For the automatic identification of norm violation, we used situations labeled by social emotions associated with norm violation (e.g., shame). We used these situations to identify the underlying social norms automatically and to categorize them into a few top-level categories. Specifically, we have used the EmpatheticDialogues dataset^24^. This is a dataset of 25 k conversations grounded in situations, each labeled according to one of 32 emotion labels.

First, we selected situations labeled by the social emotions of embarrassment, shame, and guilt (N = 4791). Next, we used GPT-3 and, for each of the situations, used the following general template for automatically generating the social norm that has been violated:

### The general template

Speaker: [Situation = “Man, when I first lost a fight, I was more humiliated that I lost than the fact that I was temporarily blind in my left eye and my head was split open with glass pieces”].

Listener: How did you feel in this situation?

Speaker: I felt [labeled emotion, e.g., ashamed].

Listener: Why did you feel [labeled emotion, e.g., ashamed]?

Speaker: I felt [ashamed] because [FIRST COMPLETION OF GPT3].

Listener: What's wrong with it? What kind of social norm did you violate?

Speaker: The social norm that I violated is [SECOND COMPLETION OF GPT3].

The code gets as an input a situation (e.g., I felt like a fool when …) and its labeled emotion (e.g., ashamed). The template is formed as a dialogue between a speaker and a listener. First, the speaker presents the situation (e.g., “Man when I first lost …”). The Listener then asks the speaker how s(he) felt in the situation, the speaker’s answer is formed in such a way that the labeled emotion is built into his answer (“I felt ashamed”), and GPT-3 is asked to complete the sentence and to explain the emotion. The GPT-3 first completion is fed back into the dialogue as a prompt and the listener asks GPT-3 to explain the social norm violated by completing the sentence “The social norm that I violated is …”. GPT-3’s second completion aims to expose the social norm violated. We run this procedure on 4971 situations with the temperature parameter set to 0.7, and *qualitatively* analyzed the outcomes of a sample of 300 situations to identify top-level categories of norms. Based on the qualitative analysis and expertise of the first author, the identified norms were:

1. **Competence**: Cognitive and personal competence. The valued individual is cognitively/personally competent (e.g., intelligent vs. fool, never give up, etc.).
2. **Politeness**: Polite and respectful. The valued individual respects the (1) boundaries, (2) property, and (3) face of the other. S(he) doesn’t cross boundaries (i.e., touching strangers), steal or use others’ property without permission or threaten the positive self-presentation of others.
3. **Trust**: Trustworthy, reliable, and honest. A valued individual is a person who can be trusted.
4. **Discipline**: Self-control of mind and body. The valued individual controls his mind, body, and behavior. She controls her body, emotions, urges, and behavior.
5. **Caring**: Caring and considerate. The valued individual cares for his valued individuals (e.g., children) by investing efforts in their survival and well-being.
6. **Agreeableness**: Agreeable, collaborative, and helpful. The valued individual collaborates with valued individuals who are not members of his closest social circle (e.g., the nuclear family).
7. **Success**: Successful. The valued individual is successful in terms of her achievements (e.g., economic or academic achievements).
8. **Conformity**: The valued individual is aware of social norms and respects social norms, specifically in the public sphere.
9. **Decency**: The valued individual associates with other valued individuals and maintains social ties with valued individuals.
10. **Loyalty**: The valued individual is loyal to her relevant social groups (e.g., a football team, ethnic group, nation)

These norms/dimensions have been translated into the following oppositional two-class categories as described in Table 1:

**Table 1:** The oppositional two class categories of the ten social norms.

Positive class	Negative class
Competent	Incompetent
Polite	Impolite
Trustworthy	Untrustworthy
Disciplined	Undisciplined
Caring	Uncaring
Agreeable	Disagreeable
Successful	Unsuccessful
Conformist	Non-conformist
Decent	Improper
Loyal	Disloyal

### Testing the zero-shot classification in identifying norm confirmation/violation

We used the EmpatheticDialogues dataset and in addition to situations labeled with guilt, embarrassment, and shame, identified situations in which the subject felt proud or grateful (N = 8886).

For example, the following situation is labeled as involving shame:

“After learning my husband of only 4 weeks was cheating on me I did the same to him. Not only was it before our marriage but I had found texts of him contacting her for sex after. To make it worse, I slept with his close family friend”.

And the following situation involves the emotion of pride:

“I am very happy to have been first over 300 students during this years at my enginering school”

Each situation has been used as an input to the zero-shot classifier. For each situation, the classifier used each of the ten dimensions by using as labels the two opposing poles of the dimension (e.g., polite vs. impolite). For example, we used as input the situation:

“After learning my husband of only 4 weeks was cheating on me I did the same to him. Not only was it before our marriage but I had found texts of him contacting her for sex after. To make it worse I slept with a close family friend of his”.

The classifier used the words “polite” and “impolite” as labels and produced the probability that the above situation should be classified as polite or impolite. We then computed as a single score the difference between the classification score of the positive class of the norm (e.g., polite) and the classification score of the negative class of the norm (e.g., impolite). This procedure resulted in ten scores corresponding with the social norms (i.e., social norms scores).

We hypothesized that if the zero-shot classifier may successfully identify norm confirmation/violation, situations labeled as proud/grateful, and hypothesized to accompany norm confirmation, should score higher on social norms than situations labeled as embarrassed, ashamed, or guilty.

We further hypothesized that situations labeled as embarrassing, ashamed, or guilty should score *below zero*, indicating that they score higher in the negative class of the norm, meaning that they involve *norm violations*.

It must be emphasized that emotions associated with a violation are negative, and those associated with norm adherence are positive. However, not all negative emotions are associated with norm violation, and not all positive emotions are associated with norm adherence. Jealousy, for instance, is a negative emotion not associated with norm violation. This is why we adhere to Sznycer’s important work and to a specific and limited set of social emotions involving social valuation vs. devaluation.

To test these hypotheses, and to avoid bias, we first removed situations in which the emotion has been explicitly mentioned in the situation. Overall, we have automatically analyzed N = 8352 situations and for each situation produced ten scores corresponding with each of the ten norms identified above (see Table 1).

The procedure is summarized as follows:

1. The input is a list of the labeled situations from the EmpatheticDialogues dataset. Each situation is labeled according to one of the social emotions (e.g., guilt)
2. For situations 1 to N and for social norms 1 to 10
3. Apply the zero-shot classifier for each situation using two labels: One indicating the positive aspect of the norm (e.g., politeness) and the other indicating the negative aspect of the norm (e.g. impoliteness)
4. Compute the difference between the probability of the norm's positive aspect and the norm's negative aspect.
5. The output is the list of labeled situations with 10 scores for each situation, each indicating the extent to which the zero-shot classifier judged them to be “norm adherence” (i.e., positive score) or “norm violation” (i.e., negative score).
6. Use the 10 scores (i.e., features) to test the hypotheses that the positive social emotions (e.g., pride) would score higher than the negative social scores (e.g., guilt).

### Results of zero-shot classification in identifying norm confirmation/violation

**Figure 1:**
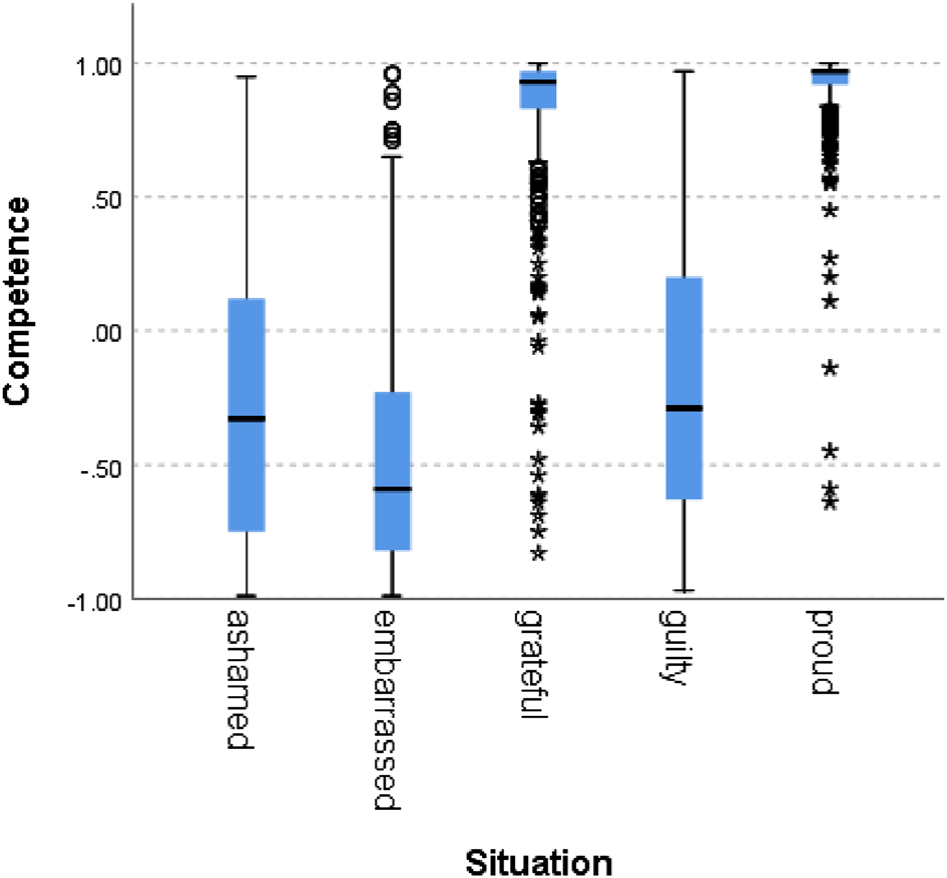
Competence scores for the different situation types.

**Figure 2:**
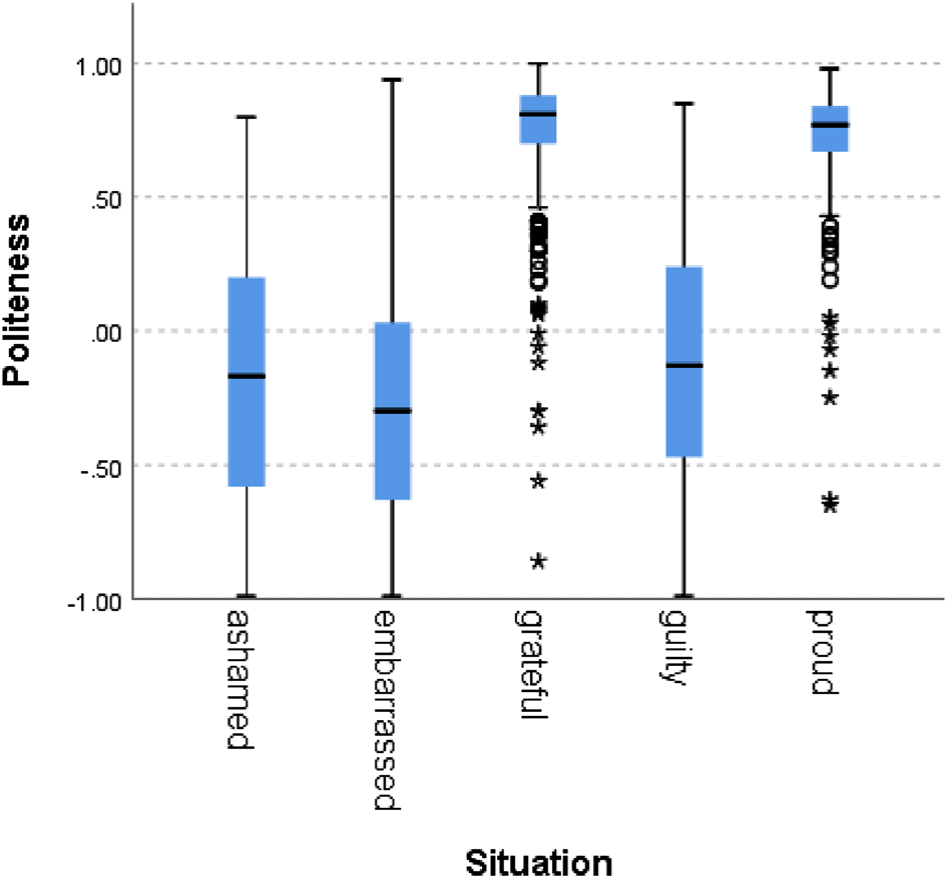
Politeness by situation.

**Figure 3:**
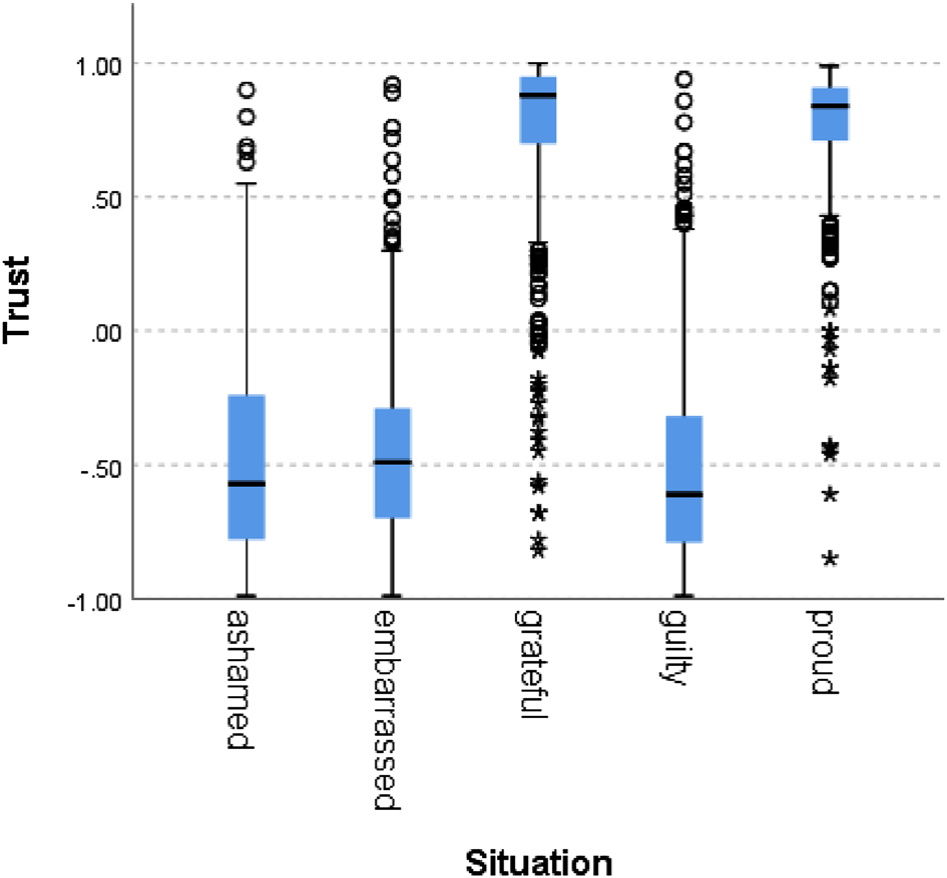
Trust by situation.

**Figure 4:**
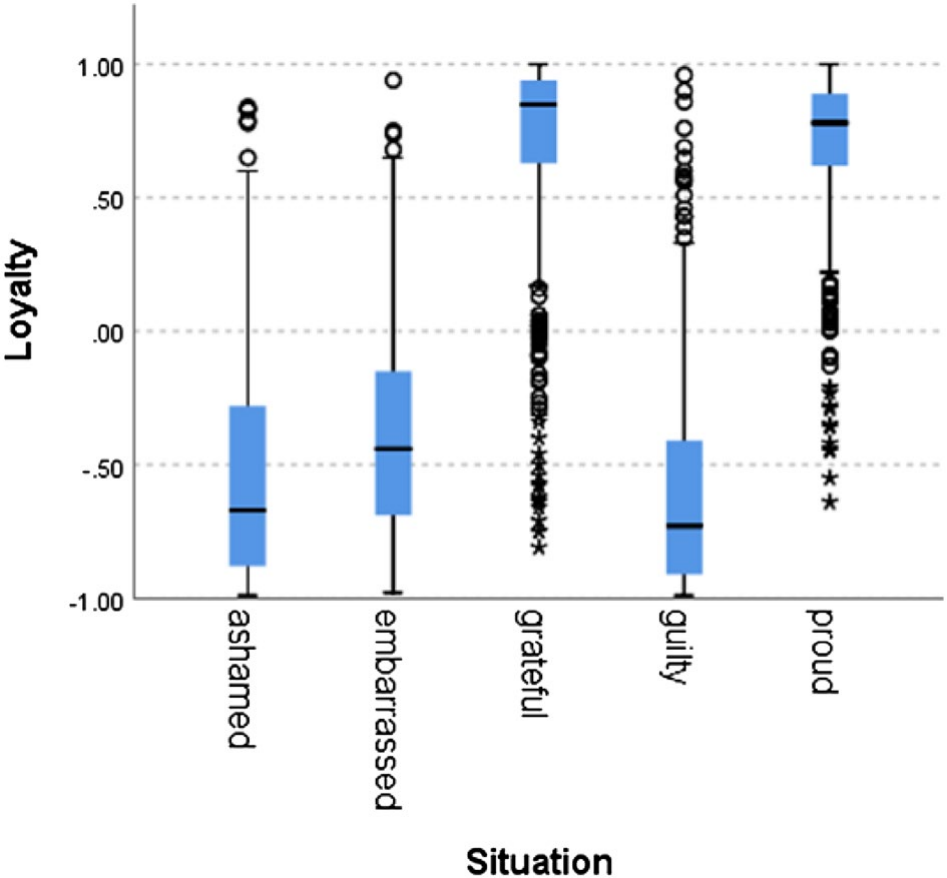
Loyalty by situation.

**Figure 5:**
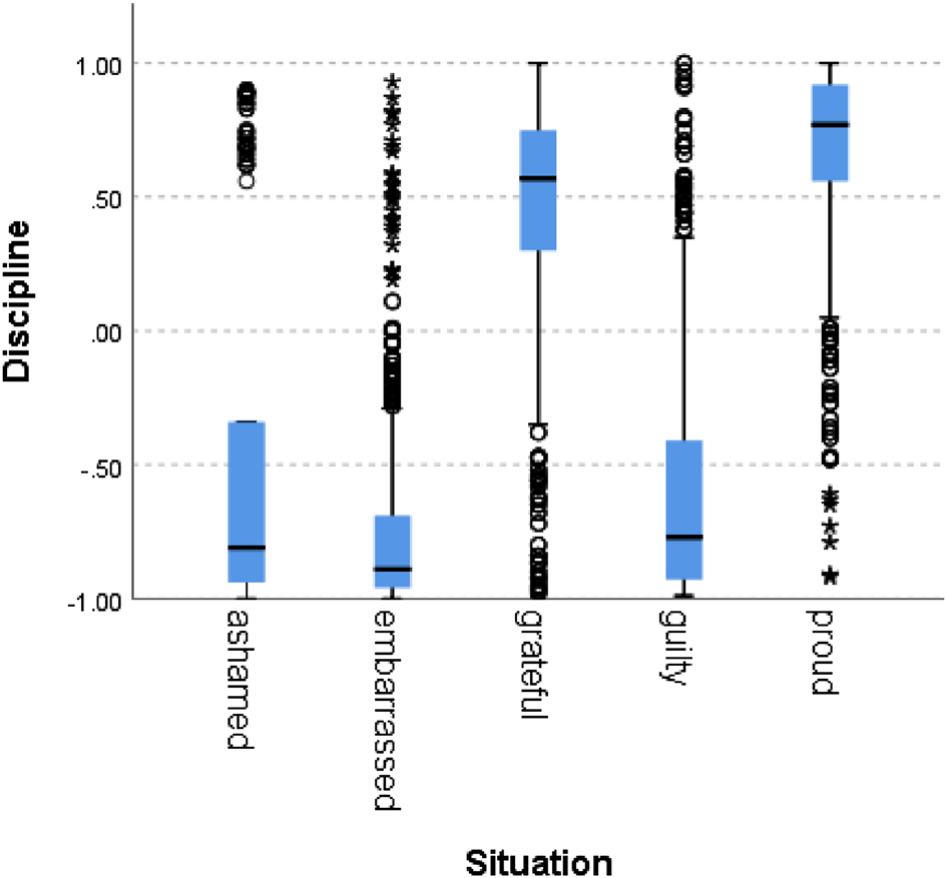
Discipline by situation.

**Figure 6:**
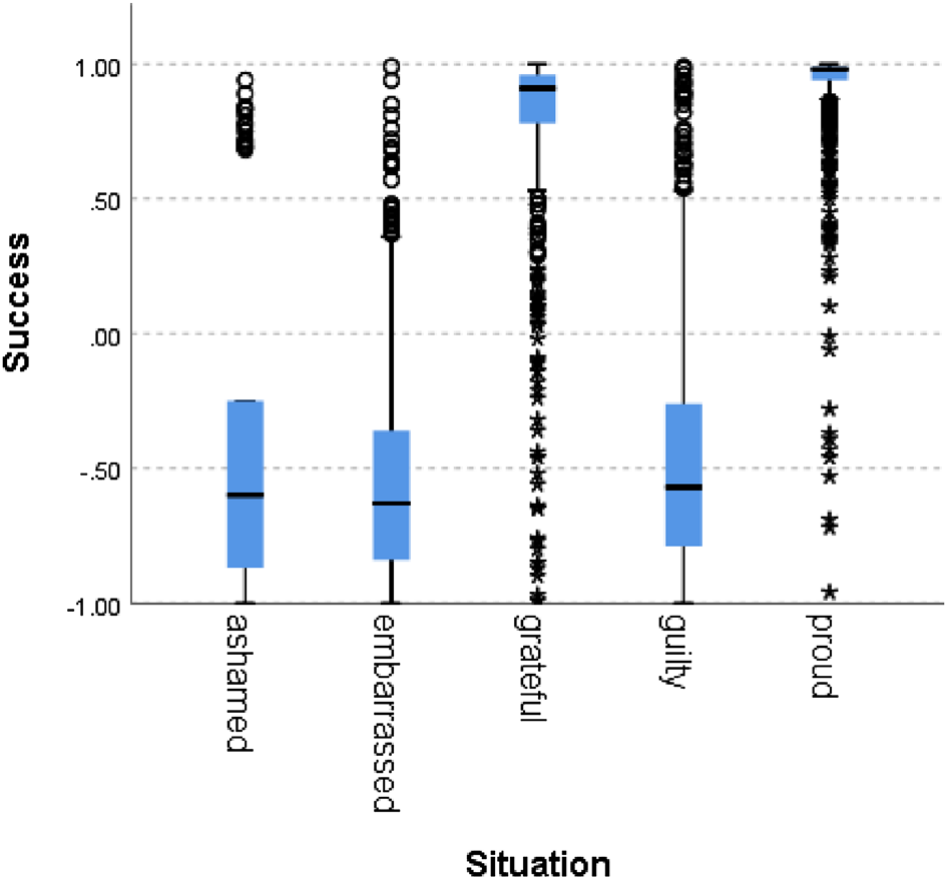
Success by situation.

**Figure 7:**
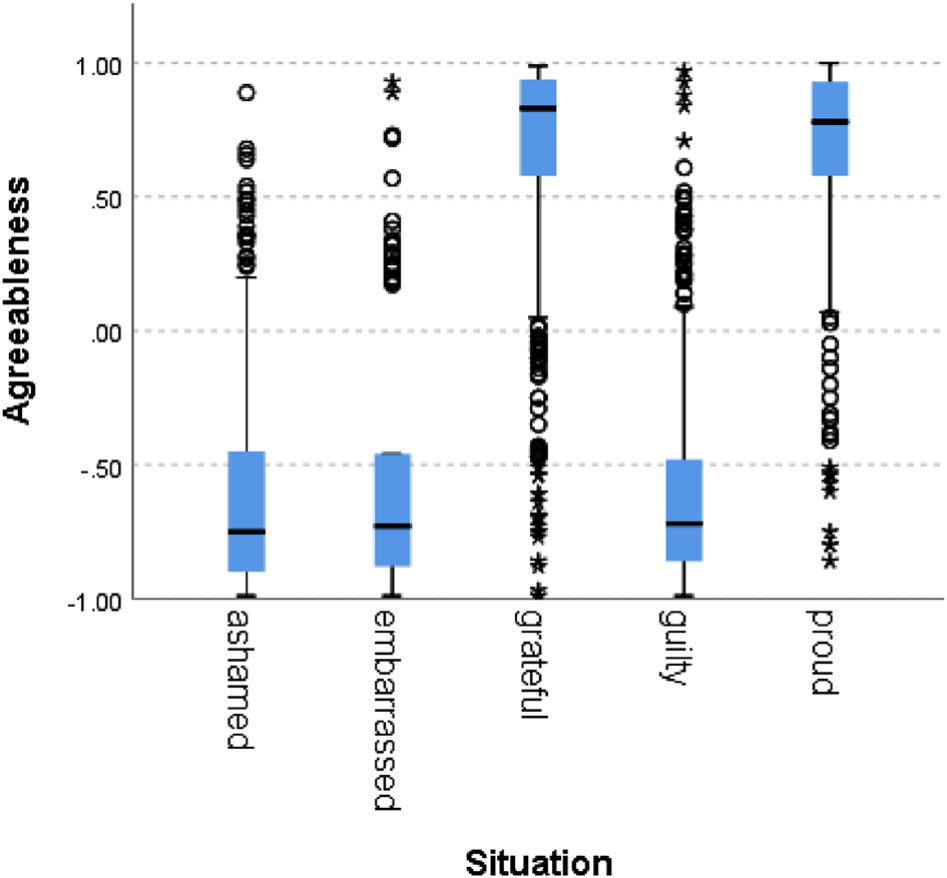
Agreeableness by situation.

**Figure 8:**
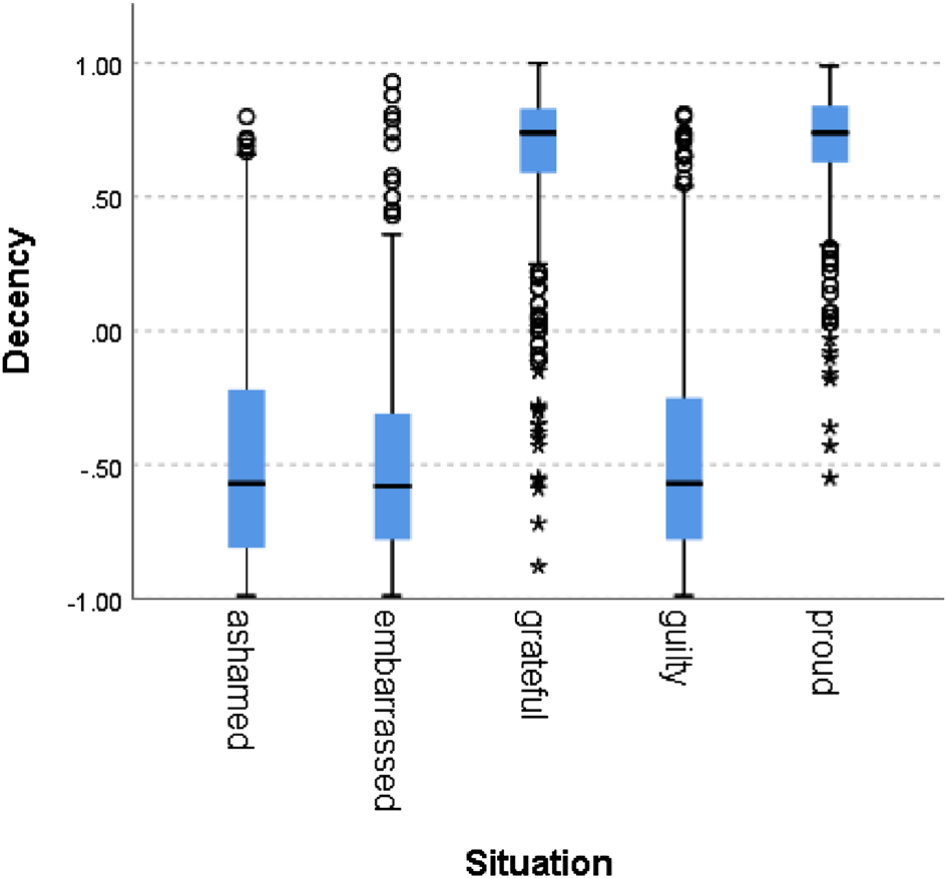
Decency by situation.

We used the Kruskal–Wallis H test to compare the ten social norms scores across situations. For example, Fig. 1 presents the results for the Competence norms:

We can see that situations labeled as proud and grateful (i.e., norm confirmation), scored higher than situations labeled with guilt, shame, and embarrassment and that situations hypothesized to indicate norm violation scored below zero, as expected. A Kruskal–Wallis H test showed a statistically significant difference in competence between the different situation types, χ^2^(4) = 5909, *p* = 0.000. Figures 2, 3, 4, 5, 6, 7, 8 present the results for the other norms:

In all of the cases, the null hypothesis has been rejected. Only in one norm (i.e., caring), the scores of the situations hypothesized to signal norm violation were not lower than 0, although the difference between the situations was statistically significant. In addition to competence, results therefore fully support our hypothesis for politeness (χ^2^(4) = 5690, *p* = 0.000), trust (χ^2^(4) = 5785, *p* = 0.000), loyalty (χ^2^(4) = 5781, *p* = 0.000), discipline (χ^2^(4) = 5271 *p* = 0.000), agreeableness (χ^2^(4) = 5621, *p* = 0.000), success (χ^2^(4) = 5737, *p* = 0.000) and decency (χ^2^(4) = 5702, *p* = 0.000). These results support the hypothesis that the zero-shot classifier can be used for identifying norm confirmation/violation.

### Automatic rule discovery and norm classification

Given the success of the zero-shot classification in identifying social emotions and norm violation, our next goal was to identify *simple rules* that may be used for classifying situations involving norm violation/confirmation.

## Methods

We grouped situations labeled with guilt, embarrassment, and shame as involving norm violation (Class 1, N = 4731) and situations labeled with pride and gratitude as involving norm confirmation (Class 0, N = 3621). To test the features' predictive power measured through the zero-shot classifier, we have applied the approach^13^ used, where first a Symbolic Classification is applied for automatic rule discovery and for identifying a simple mathematical rule for classification. Then this mathematical rule (i.e., model) is used for producing a single feature for a CRT machine learning classifier tested on other datasets. Different ML classifiers can be used, and some of them (e.g., Boosting classification) are known to produce even better results than our chosen classifier. However, we chose CRT for several reasons. First, and as we explain in this paper, we focus on identifying simple predictive models rather than gaining the best results. Second, classification and regression trees are known to be simple and powerful^25^, so they are a natural choice. Third, as our approach follows^13^’s proposal, and as they also used CRT, we chose this model to allow continuity with their approach.

This approach has been inspired by the successful venture in natural science to automatically identify simple rules (i.e., equations) governing the behavior of systems^11,26–28^ and the idea of “distilling free-form natural laws from experimental data” (the title of^11^ paper). We attempted to identify simple rules for classifying cases involving norm violation. We used HeuristicLab’s Optimizer 3.3.16^29^ to perform a symbolic classification analysis ^28^ with a maximum symbolic expression length of 5 and a maximum symbolic tree depth of 5. We have used the following features:

1. The scores of the ten social emotions (see Appendix 1 for the list of emotions),
2. The ten social norms scores,
3. The sentiment score of the situation as measured through the difference between the positive and negative scores produced by RoBERTa sentiment analysis tool^30,31^ (variable: “NegativeSentiment”).
4. A new feature, “bad”, is calculated as the difference between the classification scores of the two oppositional classes {bad, good}.

This set of features included 22 features and it is titled “the full set of features”.

By the system’s default, 66% of the dataset was used to train the model and the rest for the test. We performed ten folds, each running for 1000 generations, by examining 99,100 solutions. The target class was “1” (i.e., norm violation).

### Results of the automatic rule-discovery and norm classification

Table 2 presents the models’ performance in terms of accuracy/precision, the mathematical function identified by the procedure, and the parameters’ values.

**Table 2:** The results of the Symbolic classification analysis.

Model	Accuracy	Precision	Function	C_0_	C_1_
1	95	97	C_0_ * shame + C_1_* anger	3.03	2.093
2	89	91	C_0_ * NegativeSentiment − C_1_	0.42	− 0.49
3	95	95	C_0_ * C_1_ * − hame	− 9.1	− 0.37
4	94	96	C_0_ * bad − C_1_	0.56	− 0.57
5	95	97	C_0_ * shame + C_1_* guilt	2.44	1.69
6	94	97	C_0_ * guilt + C_1_* embarrassment	1.96	1.98
7	94	97	C_0_ * guilt + C_1_* embarrassment	2.00	1.96
8	95	97	C_0_ * shame + C_1_* guilt	2.45	1.69
9	94	97	C_0_ * guilt + C_1_* embarrassment	2.00	1.96
10	94	97	C_0_ * guilt + C_1_* embarrassment	1.99	1.98

Accuracy is presented for the test set. Precision is for the target class “1”. Precision concerns the Positive Predictive Values and Recall the True Positive Rate. All performance results in this study are in rounded percentages.

On average, the models gained 94% accuracy and 96% precision. It can be interestingly observed that most models involve the use of *social emotions* as their features/variables and that the measurement of the norms plays no role in the final models. As models 5 & 8, 9 & 10, and 6 & 7, are not different from each other, and for gaining scientific parsimony, we removed models 7, 8, and 10 from the analysis and remained with seven models only.

For testing the validity of the models and their generality beyond the specific dataset, we followed the approach of^13^ for the model’s cross-domain validation and used two different massive datasets. It must be emphasized that in this paper, we seek to identify (1) theoretically grounded (2) simple, and (3) interpretable models for the automatic identification of norm violation. Therefore, the tests presented in the next sections do not focus on performance measures per se.

In sum, we:

- Grouped the situations into two classes: one indicating norm violation and one indicating norm adherence.
- We used a Symbolic Classification procedure to generate simple models (i.e., rules) that classify situations into two classes by using the scores (i.e., features) described in the previous section.
- Each model formed through the automatic rule discovery procedure generates a score indicating whether the situation represents norm violation or norm adherence.
- Use this single score, produced by each rule, as a feature in another machine-learning model, and test the performance of these rule-generated scores to predict norm adherence/violation.

### The major experiments: testing the predictive power of the models

#### Experiment 1

We first used the Moral Stories^32^ dataset to test the previously discovered models. Moral Stories is a “crowd-sourced dataset of structured, branching narratives for the study of grounded, goal-oriented social reasoning” (ibid, p. 698). The dataset includes narratives structured as a context (composed out of the norm, the situation, and the intention of the subject), an action that follows a normative path with its consequences, and an action that follows a “divergent path” with its consequences.

For example:

Context

Norm: You should not help companies generate pollution.

Situation: Sally is a highly trained biologist.

Intention: Sally wants a prestigious job

Normative path:

Action: Sally goes to work for a company developing technologies for sustainable agriculture.

Consequence: Sally may help fight pollution caused by industrial agriculture.

Divergent path:

Action: Sally goes to work for a company producing chemical fertilizers

Consequence: The fertilizers pollute the land.

As normative actions correspond with a social norm and as divergent actions express the violation of the norm, we tested the models previously identified by using them to classify actions labeled as norm confirmation vs. norm violation. As input, we used the action (normative vs. non-normative). Each labeled action was processed according to the procedure previously described, and for each action, a score has been produced by each of the seven models. In other words, we used the zero-shot classifiers, produced measurements for the features, used the models, and for each action produced *seven* new scores corresponding with each of the models. In other words, the algorithm used the action as the input. It produced seven scores, each indicating whether the action should be classified as involving norm violation or confirmation. Overall we analyzed 12,000 actions labeled “1” (i.e., norm violation) vs. 12,000 labeled “0” (i.e., norm confirmation).

In sum:

- The input is an action labeled as “normative” or “non-normative.”
- For each action, we apply each of the rule-based models and generate for each model a single score indicating whether the action involves norm adherence or norm violation.
- Finally, we used the score produced by each model as a feature in another ML model.

#### Results of experiment 1

First, we measured the reliability of the models using Cronbach’s Alpha. Cronbach’s Alpha scored 0.73, indicating that the models produced for the EmpatheticDialogues dataset gain moderate “inter-judge” reliability when applied to another dataset. We used a Classification and Regression Tree decision model (CRT) (Using IBM SPSS), with the ranked score of each model (e.g., Model 1) as the *only* feature in the CRT machine learning model. In other words, we attempted to predict whether an action involves norm violation by using a single feature (i.e., the score produced by a model), and for prediction/classification, we used the CRT machine learning (ML) model. In addition, for each analysis, we used ten-fold cross-validation. Given the baseline of 50% of the actions labeled as “violation”, we hypothesized that if the models/rules automatically discovered are valid, then even a single feature produced through the model may be used to classify the cases beyond the baseline. The results of the analysis are presented in Table 3:

**Table 3:** The results of the CRT in classifying norm violation.

Model	Accuracy	Precision	Recall
1	70	68	76
2	68	74	66
3	67	66	67
4	73	73	73
5	66	74	64
6	66	71	65
9	66	73	63
Average	68	71	68

We can see that all models improved prediction over the baseline with an average of 68% accuracy, 71% recall, and 68% precision. When using the full set of features as features in the CRT model, we gained 78% accuracy, 81% recall, and 77% precision. When fitting a Backward Binary Logistic Regression model to the data, with the full set of features as input variables, we gained a statistically significant model (χ^2^(18) = 10,541, *p* = 0.000) with 78% accuracy, 83% recall, and 75% precision, but with 18 features (out of 22) included in the final model. It is quite remarkable that simple models identified through automatic rule discovery and involving only four social emotions, one sentiment measurement, and one score indicating whether the situation is “good” or “bad” may be used for generating a single feature that competes with the results of the Binary Logistic Regression with its model fit and 18 features. Similar results to those of the CRT were gained using a Boosting Classification model with all of the previously mentioned features (e.g., the social emotions scores). The model gained 79% accuracy, 78% precision, and 82% recall. However, we emphasized again that our aim is not to present the best classification performance but to show that simple models using a few theoretically grounded features may substantially improve the automatic identification of norm violation.

### Experiment 2

For a second validation of the models, we used the SOCIAL-CHEM-101 Dataset^33^, where 104 k real-life situations were identified and processed through crowdsourcing. More specifically, we analyzed actions (e.g., *run the blender at 5 am*) that appear in the dataset and are labeled as “legal” or “illegal”. As the percentage of illegal actions was small, we identified all actions labeled as illegal (N = 5934) and matched them with a set of N = 5934 unique actions labeled as legal. For each illegal entry, we matched a unique legal entry by using several parameters that characterize the action. For example, the dataset includes the column of “moral foundations” {i.e., care-harm, fairness-cheating, loyalty-betrayal, authority-subversion, sanctity-degradation}. Therefore, for an illegal action labeled as “care harm” for instance, we matched a legal case with the same category of moral foundation.

As illegal actions indicate that a social norm has been violated, we asked whether the models can be used for classifying an action as illegal (class “1”) or legal (class “0”). Each action has been subjected to the analysis of the relevant features, and using the features, the models have been applied to produce seven scores for each action.

In sum:

- The input was an action labeled as legal or illegal.
- For each action, we have applied the seven rule-based models and generated a single score indicating whether the action involves norm adherence or norm violation.
- The scores produced by the rule-based models were used as features for another ML model that classified the action as norm violation or norm adherence.

#### Results of experiment 2

We first computed the scores produced by the seven models. Their Cronbach’s Alpha score was 0.93, indicating that the seven models highly agreed. We gained 82% accuracy, 87% recall, and 80% precision using the scores of the seven models as features in a CRT ML model and the same procedure as applied before. The detailed performance of the seven models appears in Table 4:with an average of 76% accuracy, 77% precision, and 78% recall. Using the full set of features in a CRT model gained 89% accuracy, 87% precision, and 84% recall.

**Table 4:** The CRT performance of the models when used for the SOCIAL-CHEM-101 Dataset.

Model	Accuracy	Precision	Recall
1	73	71	76
2	70	68	77
3	77	73	87
4	77	86	73
5	80	85	77
6	77	76	77
9	77	77	77
Average	76	77	78

### Identifying which norm has been violated

For testing our ability to identify *which* norm has been violated, we used the SOCIAL-CHEM-101 dataset and cases labeled as illegal. Following Graham et al., (2013), SOCIAL-CHEM-101 labels the situations according to five moral norms. We have identified cases where the situations were labeled only according to one of these norms. It is important to emphasize that our algorithm is generic and can test the violation of different norms. For measuring the norm violations of the Social Chemistry dataset, we have used the norm proposed by the researchers who developed this dataset. Previously, we have used the norms identified through the GPT-3. Our algorithm is general enough to include different social and moral norms.

The distribution of these moral norms in SOCIAL-CHEM-101 appears in Table 5:

**Table 5:** Frequency of the moral norms in SOCIAL-CHEM-101.

Norm	Frequency	Percent
Care ⇄ Harm	2024	50.5
Fairness ⇄ Cheating	1070	26.7
Loyalty ⇄ Betrayal	30	0.7
Authority ⇄ Subversion	508	12.7
Sanctity ⇄ Degradation	375	9.4
Total	4007	100

Given the extremely low prevalence of the loyalty norm, it has been removed from the analysis, and the moral norms that we analyzed were care (50.9%), fairness (26.9%), authority (12.8%), and sanctity (9.4%). For identifying which norm has been violated, we used a Boosting Classification ML model with five-fold cross-validation and the full set of features as previously presented (e.g., the norm violation scores).

### Results of violation identification

The model performance in identifying the violated norm is presented in Table 6:

**Table 6:** Performance measures in identifying the violated moral norm.

Norm	Baseline	Recall	Precision	Improvement
Care	51	90	75	39
Fairness	27	71	71	44
Authority	13	42	78	29
Sanctity	9	31	53	22

We can think about the improvement in prediction in terms of a random guess with the limited knowledge of the norm’s prevalence in the dataset; for instance, knowing that the care dimension is approximately 51% of the dataset, we could have randomly guessed in 51% of the cases that the violated norm is the one of caring. In this context, an improvement may be conceived as an improvement in prediction over the baseline.

On average, there was a 34% improvement in prediction. The five features with the highest influence in the model were four of the norm violation measures: (1) trust, (2) caring, (3) loyalty, and (4) conformist, and one social emotion (anger). This improvement in prediction indicates that norm violation may be automatically identified and that the exact type of the violated norm can also be identified using the social norm scores and the social emotion measures as computed through the zero-shoot classification procedure.

### Ethical approval

All methods were carried out by relevant guidelines and regulations.

## Discussion

Social norms are either descriptive, representing the prevalence of a certain behavior (e.g., avoiding tax payment), or injunctive, representing the extent to which the behavior is approved by a relevant reference group. As social animals, human beings are specifically sensitive to the valuation of others and hence to injunctive norms and their violation, which is accompanied by social emotions such as guilt (e.g., ^34^). While social norms may be culturally specific and cover numerous informal “rules”, how people *respond* to norm violation through evolutionary-grounded social emotions may be much more general and provide us with cues for the automatic identification of norm violation.

In this paper, we have developed and tested several models for automatically identifying the violation of social norms. One would hardly find papers dealing with the automatic identification of *social* norm violations. Those focusing on norm violation usually concern norms of interaction in specific communities such as Reddit^35,36^. This scarcity of research in identifying and recognizing social norm violations points to the fact that the automatic identification of social norm violations is an open challenge.

We have shown that through (1) the measurement of social emotions and social norms in textual data (2) zero-shot classification, (3) the use of the measured features in the automatic-rule discovery algorithm, and (4) the use of the discovered rules for generating simple features used in a Decision Tree model, may provide substantial improvement in the prediction of social norm violation. Moreover, using GPT-3, and domain expertise, we were able to identify top-level categories of social norms. Using the top-level categories of social norms, we were able to correctly identify the exact norm that has been violated. However, the number of social norms that we tested in the dataset was limited. The paper is, therefore another instance in which modern large language models, such as GPT-3, as combined with the domain expertise of a discipline (e.g., psychology), may advance research in psychology, the social sciences, and the humanities^37^. Our paper presents some preliminary results but will be developed in future studies to include the identification of norm violation in conversation, the use of multi-modality for social norm violation, and the use of large language models to identify culturally specific norms.

Our study is limited to developing tools for identifying the violation of “general” social norms. However, the granularity level of norms may span from social groups to the individual level. In this context, it was argued that “people have preferences for following their ‘personal norms’ what they believe to be the right thing to do”^38^^, p. 2^ and that personal norms may be a powerful explanatory idea in understanding human unselfish behavior. This idea may be tested using tools of computational personality analysis^39^ where relatively stable patterns of thought, emotion, and behavior (i.e., personality) may be extended to include personal norms and the tendency to follow them. For instance, the representation of others (i.e., beliefs about others), is considered to be a major dimension in understanding human personality and it has been measured through novel tools of AI for a better understanding of fictional characters^40^. In fact, and in the context of personal norms^38^, suggest that AI may be used “to better navigate the complex landscape of human morality and to better emulate human decision-making” (ibid, p. 11) even in contexts governed in the past by different methodologies such as the behavioral economics. One may hypothesize, for instance, that a person holding negative paranoid beliefs of others and following conspiracy theories may be less prone to express one-shot anonymous unselfishness when he considers his interlocutor to belong to out-group players. It is possible to measure the variability of norms using AI and language-based models tools. As explained by^41^^, p. 2^ “what matters is not just the monetary payoffs associated with actions, but also how these actions are described”. For example, automatically analyzing a massive amount of textual data following Hurricane Catarina could have tested whether cooperative actions are described differently by people with different moral norms. It could have been hypothesized that those with more negative beliefs about other out-group members are more inclined to hold different norms. This hypothesis aligns with^42^ findings. It may suggest that those individuals may also describe cooperative actions in a less favorable language and may be more inclined to behave less cooperatively (e.g. fewer donations for charity outside their reference group). In addition, our paper is limited by the inevitable choice of datasets, theoretical approaches, and ML models. Therefore, the results presented here are preliminary and should be modestly limited to the specific context of this study.

## Data Availability

Data sets are available here: https://drive.google.com/file/d/1AX8_1W2AubqbAQgbusucRvEB_kFKWSt4/view.

## Appendix 1

The social emotions:

1. Embarrassment
2. Shame
3. Guilt
4. Jealousy
5. Envy
6. Empathy
7. Pride
8. Anger
9. Gratitude
10. Sadness

## References

[1] APA Dictionary of Psychology. Social norm. Available from: https://dictionary.apa.org/social-norm. Accessed 12 May 2023.

[2] Eriksson K, Strimling P, Gelfand M, Wu J, Abernathy J, Akotia CS (2021). Perceptions of the appropriate response to norm violation in 57 societies. Nat. Commun..

[3] Borat. In: Wikipedia [Internet]. Wikimedia Foundation; 2023 [cited 2023 May 12]. Available from: https://en.wikipedia.org/wiki/Borat.

[4] Gross J, Vostroknutov A (2022). Why do people follow social norms?. Curr Opin Psychol..

[5] van Kleef GA, Gelfand MJ, Jetten J (2019). The dynamic nature of social norms: New perspectives on norm development, impact, violation, and enforcement. J. Exp. Soc. Psychol..

[6] Defense Advanced Research Projects Agency (DARPA). DARPA announces AI Next campaign. 2021 May 3. Available from: https://www.darpa.mil/news-events/2021-05-03a. Accessed 12 May 2023.

[7] OpenAI. GPT-3: Language models are few-shot learners. 2020. Available from: https://github.com/openai/gpt-3. Accessed 12 May 2023.

[8] Brown T, Mann B, Ryder N, Subbiah M, Kaplan JD, Dhariwal P (2020). Language models are few-shot learners. Adv. Neural Inf. Process. Syst..

[9] Wolf, T., *et al.* BART-Large-MNLI. Hugging Face; 2020. Available from: https://huggingface.co/facebook/bart-large-mnli. Accessed 12 May 2023.

[10] Yin. W., Hay. J., & Roth, D. Benchmarking zero-shot text classification: Datasets, evaluation and entailment approach. Preprint at arXiv:1909.00161. 2019 Sep 1. Available from: https://arxiv.org/abs/1909.00161. Accessed 12 May 2023.

[11] Schmidt M, Lipson H (2009). Distilling free-form natural laws from experimental data. Science.

[12] Curry OS, Mullins DA, Whitehouse H (2019). Is it good to cooperate? Testing the theory of morality-as-cooperation in 60 societies. Curr. Anthropol..

[13] Neuman Y, Cohen Y (2022). Predicting change in emotion through ordinal patterns and simple symbolic expressions. Mathematics.

[14] Sznycer D (2022). Value Computation in Humans.

[15] Sznycer D, Sell A, Lieberman D (2021). Forms and functions of the social emotions. Curr. Dir. Psychol. Sci..

[16] Billig M (2005). Laughter and Ridicule.

[17] Bas-Hoogendam, J. M., van Steenbergen, H., Kreuk, T., Van der Wee, N.J., Westenberg, P.M. How embarrassing. 10.1371/journal.pone.0176326 PMC5404760 28441460

[18] van Kleef GA, Wanders F, Stamkou E, Homan AC (2015). The social dynamics of breaking the rules: Antecedents and consequences of norm-violating behavior. Curr. Opin. Psychol..

[19] Robertson TE, Sznycer D, Delton AW, Tooby J, Cosmides L (2018). The true trigger of shame: Social devaluation is sufficient, wrongdoing is unnecessary. Evol. Hum. Behav..

[20] Vaish A (2018). The prosocial functions of early social emotions: the case of guilt. Curr. Opin. Psychol..

[21] Shen, J., *et al.* TaxoClass: Hierarchical multi-label text classification using only class names. In *Proceedings of the 2021 Conference of the North American Chapter of the Association for Computational Linguistics: Human Language Technologies*; 2021. p. 4239–4249.

[22] Authors. Identifying social norm violation through zero-shot classification: From Borat to American Pie. Under review; (2022).

[23] Sznycer D, Lukaszewski AW (2019). The emotion–valuation constellation: Multiple emotions are governed by a common grammar of social valuation. Evol. Hum. Behav..

[24] Rashkin, H., Smith, E. M., Li, M., & Boureau, Y. L. Towards empathetic open-domain conversation models: A new benchmark and dataset. Preprint at arXiv:1811.00207; (2018).

[25] Krzywinski M, Altman N (2017). Classification and regression trees. Nat Methods..

[26] Guimerà R, Reichardt I, Aguilar-Mogas A, Massucci FA, Miranda M, Pallarès J, Sales-Pardo M (2020). A Bayesian machine scientist to aid in the solution of challenging scientific problems. Sci. Adv..

[27] Udrescu SM, Tegmark M (2020). AI Feynman: A physics-inspired method for symbolic regression. Sci. Adv..

[28] Wagner S, Kronberger G, Beham A, Kommenda M, Scheibenpflug A, Pitzer E, Vonolfen S, Kofler M, Winkler S, Dorfer V, Klempous R, Nikodem J, Jacak W, Chaczko Z (2014). Architecture and design of the HeuristicLab optimization environment. Advanced Methods and Applications in Computational Intelligence.

[29] HeuristicLab. https://dev.heuristiclab.com. Accessed May 12, 2023.

[30] https://huggingface.co/finiteautomata/bertweet-base-sentiment-analysis. Accessed May 12, 2023.

[31] Pérez, J. M., Giudici, J. C., & Luque, F. pysentimiento: A python toolkit for sentiment analysis and social NLP tasks. Preprint at arXiv:2106.09462 (2021).

[32] Emelin, D., Bras, R. L., Hwang, J. D., Forbes, M., & Choi, Y. Moral stories: Situated reasoning about norms, intents, actions, and their consequences. Preprint at arXiv:2012.15738 (2020).

[33] Forbes, M., Hwang, J. D., Shwartz, V., Sap, M., & Choi, Y. Social chemistry 101: Learning to reason about social and moral norms. Preprint at arXiv:2011.00620 (2020).

[34] Jacobson RP, Jacobson KJ, Reid AE (2021). Guilt enhances the persuasive effects of injunctive but not descriptive social norms. Soc. Psychol. Personal. Sci..

[35] Chandrasekharan E, Samory M, Jhaver S, Charvat H, Bruckman A, Lampe C (2018). The Internet's hidden rules: An empirical study of Reddit norm violations at micro, meso, and macro scales. Proc. ACM Hum. Comput. Interact..

[36] Park, C. Y., *et al.* Detecting community sensitive norm violations in online conversations. arXiv:2110.04419 (2021).

[37] Neuman Y, Danesi M (2022). Interpreting through AI: A note on the possibility of weaving ancient traditions with novel technologies. Dig. Scholarsh. Humanit..

[38] Capraro V, Perc M (2021). Mathematical foundations of moral preferences. J. R. Soc. Interface.

[39] Neuman Y (2016). Computational Personality ANALYSIS: Introduction, practical Applications and Novel Directions.

[40] Neuman Y, Danesi M, Vilenchik D (2022). Using AI for Dialoguing with Texts: From Psychology to Cinema and Literature.

[41] Capraro, V., Halpern, J. Y., & Perc, M. From outcome-based to language-based preferences. Preprint at arXiv:2206.07300 (2022).

[42] Graham J, Haidt J, Nosek BA (2009). Liberals and conservatives rely on different sets of moral foundations. J. Pers. Soc. Psychol..

